# A Novel Splice-Site Mutation in *ALS2* Establishes the Diagnosis of Juvenile Amyotrophic Lateral Sclerosis in a Family with Early Onset Anarthria and Generalized Dystonias

**DOI:** 10.1371/journal.pone.0113258

**Published:** 2014-12-04

**Authors:** Saima Siddiqi, Jia Nee Foo, Anthony Vu, Saad Azim, David L. Silver, Atika Mansoor, Stacey Kiat Hong Tay, Sumiya Abbasi, Asraf Hussain Hashmi, Jamal Janjua, Sumbal Khalid, E. Shyong Tai, Gene W. Yeo, Chiea Chuen Khor

**Affiliations:** 1 Institute of Biomedical and Genetic Engineering (IBGE), Islamabad, Pakistan; 2 Human Genetics, Genome Institute of Singapore, A*STAR, Singapore, Singapore; 3 Department of Cellular and Molecular Medicine and Institute for Genomic Medicine, University of California at San Diego, La Jolla, California, United States of America; 4 Ali Medical Center, F8/1, Islamabad, Pakistan; 5 Signature Research Program in Cardiovascular & Metabolic Disorders, Duke-NUS Graduate Medical School, Singapore, Singapore; 6 Department of Pediatrics, Yong Loo Lin School of Medicine, National University of Singapore, Singapore, Singapore; 7 International Islamic University, Islamabad, Pakistan; 8 College of Physicians and Surgeons (CPSP), Islamabad, Pakistan; 9 Department of Medicine, Yong Loo Lin School of Medicine, National University of Singapore, National University Hospital System, Singapore, Singapore; 10 Saw Swee Hock School of Public Health, National University of Singapore, National University Hospital System, Singapore, Singapore; 11 Department of Physiology, Yong Loo Lin School of Medicine, National University of Singapore, Singapore, Singapore; Inserm, France

## Abstract

The diagnosis of childhood neurological disorders remains challenging given the overlapping clinical presentation across subgroups and heterogeneous presentation within subgroups. To determine the underlying genetic cause of a severe neurological disorder in a large consanguineous Pakistani family presenting with severe scoliosis, anarthria and progressive neuromuscular degeneration, we performed genome-wide homozygosity mapping accompanied by whole-exome sequencing in two affected first cousins and their unaffected parents to find the causative mutation. We identified a novel homozygous splice-site mutation (c.3512+1G>A) in the *ALS2* gene (NM_020919.3) encoding alsin that segregated with the disease in this family. Homozygous loss-of-function mutations in *ALS2* are known to cause juvenile-onset amyotrophic lateral sclerosis (ALS), one of the many neurological conditions having overlapping symptoms with many neurological phenotypes. RT-PCR validation revealed that the mutation resulted in exon-skipping as well as the use of an alternative donor splice, both of which are predicted to cause loss-of-function of the resulting proteins. By examining 216 known neurological disease genes in our exome sequencing data, we also identified 9 other rare nonsynonymous mutations in these genes, some of which lie in highly conserved regions. Sequencing of a single proband might have led to mis-identification of some of these as the causative variant. Our findings established a firm diagnosis of juvenile ALS in this family, thus demonstrating the use of whole exome sequencing combined with linkage analysis in families as a powerful tool for establishing a quick and precise genetic diagnosis of complex neurological phenotypes.

## Introduction

Childhood neurological disorders comprise a diverse group of diseases with overlapping clinical presentation across disease subgroups and extreme variable expressivity within subgroups, making it very challenging to establish a precise diagnosis. Several neurological conditions also require an extensive set of tests for diagnostic workup including imaging, neurophysiological and tissue sampling that may be both invasive and expensive. Next-generation sequencing provides new opportunities to overcome these challenges in establishing a quick and accurate diagnosis [1], [2]. We carried out a study on a large consanguineous Pakistani family presenting with a severe childhood-onset neurological phenotype without knowing the specific disease to test the power of next generation sequencing in establishing diagnosis.

## Materials and Methods

### Subjects

**Figure 1:**
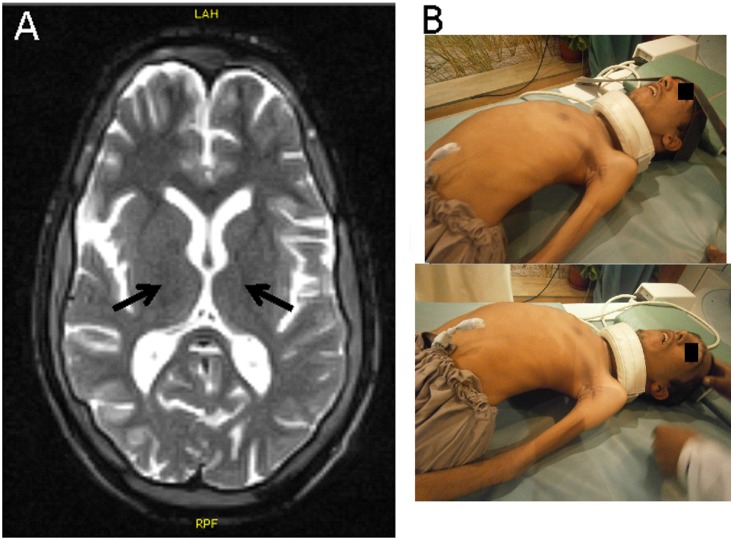
Patient characteristics. a) MRI brain axial T2 weighted sequence showing thinning of the corticospinal tracts (black arrows indicating corticospinal tracts) and b) photographs of one of the patients (IV-4) showing severe scoliosis.

The clinical characteristics of the patients are described in the results section. One subject (IV-4) was examined by magnetic resonance imaging (MRI) of both the brain and cervical spine (Figure 1).

### Ethics statement

This study was approved by the institutional review board of the Institute of Biomedical and Genetic Engineering (IBGE), Islamabad, Pakistan. All participants are adults and gave written informed consent to participate in this study. The brother of IV-4 gave written informed consent (as outlined in PLOS consent form) to publish these case details.

### Genotyping

**Figure 2:**
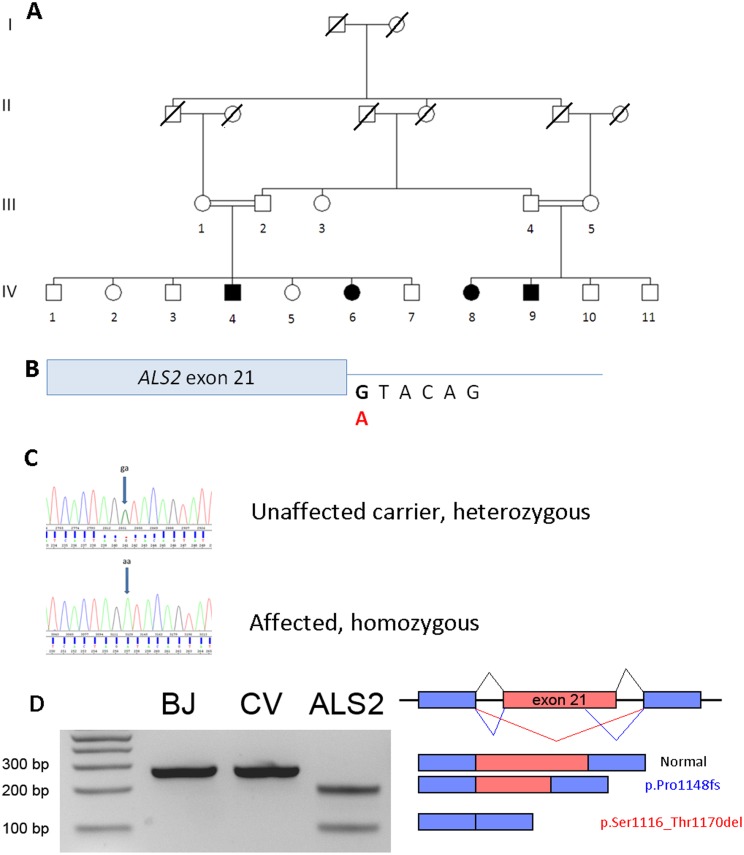
Genetic analysis of pedigree. a) Pedigree examined in this study. Only individuals from generations III and IV were available for genetic analysis. b) Location of the mutation NM_020919.3:c.3512+1G>A at the boundary of the 21st intron of the *ALS2* gene and c) confirmation by Sanger sequencing. d) RT-PCR of total RNA isolated from patient (IV-4 labeled as ALS2) and two control fibroblast cells (labeled BJ and CV), visualized on a gel alongside an Invitrogen 1 kb+ ladder. Three splicing transcripts corresponding to the three bands (102 bp encoding p.Ser1116_Thr1170del [red], 200 bp encoding p.Pro1148fs [blue] and 267 bp encoding the normal protein [black]) were confirmed by Sanger sequencing and illustrated in the figure.

Genomic DNA was isolated from whole blood from 16 individuals from generations III and IV of the family (Figure 2a) and genotyped on the Illumina OmniExpress v1.1 BeadChip array for a total of 729,698 genetic markers. After removal of single nucleotide polymorphisms (SNPs) that were monomorphic or failed genotyping in >1 sample, 443,914 genome-wide SNP markers remained for analysis. We confirmed the reported familial relationships among genotyped samples using PLINK identity by descent analysis (–genome) [3]. We then scanned the data for large homozygous segments (>5 Mb) that are shared among affected individuals but not unaffected individuals as well as for statistical evidence of linkage of genomic regions to the disease. Homozygosity mapping was conducted using PLINK v1.07 using the default settings [3]. Parametric linkage analysis was performed using Simwalk 2.19 assuming autosomal recessive inheritance of the disease and 100% penetrance [4], [5].

### Whole exome sequencing

Targeted enrichment was performed on 1 µg of genomic DNA from each individual (two affected first cousins and one unaffected parent from each) using the Nimblegen SeqCap EZ Exome v3 kit and barcoded for sequencing on a single lane of a multiplexed 2×101 bp sequencing run on the Illumina HiSeq 2000 platform. Each individual was sequenced to a high mean coverage of 65–74 reads per target base, with 96% of the target exome covered by 10 or more reads. Reads were mapped using BWA v1.7 [6] and variants were called using the GATK v2 Unified Genotyper following the recommended guidelines by GATK ‘Best practices for variant calling v3’ [7]. We used the following primer sequences to validate detected mutations in ALS2 c.3512+1G>A and GARS p.Gly546Glu by polymerase chain reaction and Sanger sequencing: ALS2-forward CAGTGAGACTGAGTATGGTTTTGG, ALS2-reverse TGCTTGTTAAATAATGATGGTGC, GARS-forward TTTACCAGCAGGCCTATTTCTG, GARS-reverse CAAAAGCTTTAAGGAGCAGTGAC.

### Reverse transcriptase polymerase chain reaction (RT-PCR)

First-strand cDNA was generated by reverse-transcribing 1 µg of total RNA isolated from fibroblast cells taken from a single patient (IV-4) homozygous for the *ALS2* mutation using oligo-dT primer and Superscript III reverse transcriptase (Invitrogen). Since we did not collect cells from unaffected family members, we also isolated RNA from the BJ foreskin fibroblast cell line (ATCC) and the Craig Venter (CV) fibroblast cell line as controls. PCR was performed for 40 cycles using ∼25 ng cDNA template and the following primer sequences to amplify the region flanking the splice junctions 5′ and 3′ of exon 21: forward AGGAAAAATGTGCGGTCAAG and reverse CCAAACTGGGTAACCACCAC. Products were visualized on a gel and confirmed by Sanger sequencing.

## Results

The disorder presented as a similar sequence of events in all affected members of the family, with an early age of presentation after birth following an uneventful pregnancy. Motor milestones were markedly delayed; the patients developed head control at approximately 10 months and had delayed crawling or creeping at about 1 year of age. IV-8 was able to walk at 4 years of age and IV-9 was able to walk after 1 year of age. IV-4 and IV-6 were never able to walk. There was rapid progression of motor weakness beginning in the lower limbs, with loss of walking ability by 6 years of age for IV-8 and by 3 years of age by IV-9. There was early onset of scoliosis associated with the muscle weakness (Figure 1b). Progression of scoliosis and weakness of the upper and lower limbs resulted in the affected individuals becoming bed-bound between 5–19 years of age. Cognitive ability appeared to be intact as the affected could understand instructions and appeared to be able to follow at least one-step commands and to indicate their needs with eye movements. They had marked dysarthria, drooling and bouts of emotional lability and uncontrolled laughter. Bladder and bowel control appeared to be normal.

Clinical examination was performed for each of these individuals when they were 20–24 years of age (Text S1 in File S1). The examination of each of these patients showed normal hearing responses and ocular movements. Cognition was found to be largely intact as the patients could keep track of time, follow basic commands and use their facial expressions and eye movements to communicate understanding of conversation. Nonetheless, we were not able to fully assess cognitive functions in these patients because they were either anarthric or severely dysarthric. There was marked thoracolumbar scoliosis. There was a paucity of facial muscle movements and marked drooling and dysarthria. Tone was increased in all 4 limbs and there was significant wasting of distal muscles in the limbs. Muscle power was grade 1 in all 4 limbs and there were markedly brisk deep tendon reflexes and upgoing plantar responses. Ankle clonus was present. There were contractures in the limbs as well as opisthotonic posturing and dystonic movements of the limbs.

Sensory and motor nerve conduction velocities (NCV’s) were tested in radial and ulnar nerves for the upper limbs and in peronial and tibial nerves for the lower limbs. The NCV’s were within normal limits. One subject (IV-4) was examined by magnetic resonance imaging (MRI) of both the brain and cervical spine (Figure 1a). MRI of the brain for IV-4 showed thinning of the corticospinal tracts. MRI of the spine was normal.

Homozygous segments of length >10 Mb accounted for 6–8% of the genome in all siblings IV1-7 and IV8-11 as well as the parents III-1 and III-5 (Figure 2a), confirming that these individuals are the offspring of consanguineous first cousin unions. We identified a single 7-million base pair region on chromosome 2q33.1 (chr2∶199,182,704-206,511,008) that was homozygous only in affected individuals of this family. Parametric linkage analysis using 55 independent markers between chr2∶180–213 Mb with Simwalk2.19 confirmed that this region showed significant linkage with the disease, with a LOD score of 3.51 [4], [5]. A total of 92 genes were present within this shared homozygous segment.

To identify the causal mutation, we performed whole exome sequencing in two affected cousins (IV-6, IV-9 and one each of their unaffected parents (III-2, III-5) to identify a set of mutations that segregate with the disease. A total of 47,134 variants were identified in these four individuals. We focused our analysis on 133 nonsynonymous mutations (including nonsense, splice-site and frame shifting indels) which were homozygous in the affected children and heterozygous in their parents (Figure 2a) and identified only 4 that occurred within the homozygous region on chr2q33.1. Three were common variants with allele frequencies greater than 5% in all HapMap and 1000 genomes populations. This left us with only one donor splice-site mutation NM_020919.3:c.3512+1G>A at the boundary of the 21st intron of the *ALS2* gene (OMIM ID 606352; Figure 2b). The consequence of this change can result in skipping of exon 21 (Alamut 2.0 splicing prediction) [8]. This mutation has not been previously reported and is not present in any of the public SNP databases (HapMap, 1000 genomes populations [9] and the NHLBI exome variant server (EVS) databases (URL: http://evs.gs.washington.edu/EVS/)). To confirm that this is the causal mutation, we performed Sanger sequencing of all 16 members of the family and demonstrated that this mutation segregated perfectly with the disease (Figure 2c).

To evaluate the effects of the splice site mutation, we performed reverse transcriptase polymerase chain reaction (RT-PCR) experiments on total RNA isolated from patient fibroblast cells (IV-4) and two normal control fibroblast cell lines (Figure 2d). The experiments confirmed that the c.3512+1G>A mutation results in skipping of exon 21, which is predicted to produce an in-frame deletion of 55 amino acids in the encoded protein (p.Ser1116_Thr1170del). We observed the presence of an alternate donor splice site within exon 21 that produces a transcript missing 67 bases encoded by exon 21 which is predicted to result in a frameshift mutation (p.Pro1148fs). While simpler statistical models such as weight matrix model (WMM), first-order markov model (MM) and maximal dependence decomposition (MDD) that compare the original (TAGgtacag; WMM:3.66; MM: 4.81; MDD:9.98 bits) with the alternative donor splice site (CTAgtatgt; WMM:4.92; MM: 4.47; MDD:8.08, with capital letters indicate exonic sequence) score the alternative splice site higher, a higher performing maximum entropy model (MAXENT) that incorporates all pair-wise dependencies indicate a similar splice site recognition strength (4.67 for the original to 4.69 bits for the alternative) [10]. This equally efficient splice site choice led to the production of two different truncated transcripts in the patient that are not present in normal individuals. Our experiments have also confirmed the absence of the normal, full-length transcript in the patient (Figure 2d).

Should we have examined all 133 variants that segregated with the disease in the four sequenced individuals, including those outside the homozygous region, and excluded the 131 common variants that are present at >1% frequencies in any HapMap and1000 genomes populations as well as the NHLBI EVS databases, we would have identified the same disease mutation and only one other nonsynonymous variant p.Ser45Tyr (predicted by the SIFT tool to be tolerated) in the trypsin2 gene *PRSS2* on chromosome 7, suggesting that in the absence of multiple samples from a large pedigree for robust linkage analysis, whole-exome sequencing followed by bioinformatic filtering may be sufficient for the rapid identification of candidate mutations for rare recessive diseases. However, given the absence of linkage in this region, the *PRSS2* p.Ser45Tyr mutation is not expected to segregate with the disease upon examination of additional family members.

Finally, we further analyzed 216 known neurological and neuromuscular disease genes [11] (Table S1 in File S1) and identified 10 nonsynonymous variants that are rare (<1%) or absent in all 1000 genomes populations or the exome variant server database (Table S2 in File S1), which includes p.Gly546Glu in the *GARS* gene mutated in autosomal dominant form of Charcot Marie Tooth disease type 2D (CMT2D) and distal hereditary motor neuropathy, type VA, as well as p.Val222Ile in the *SH3T2C* gene mutated in Charcot-Marie-Tooth disease, type 4C [12]. With the exception of *ALS2* mutation, none of these other variants lie in linked/homozygosity regions and none segregated with the disease in sequenced individuals of this family, suggesting that none of them were causal mutations of neurological phenotypes in this family. Given that these mutations lie in highly conserved regions, sequencing of a single proband without linkage information might have led to mis-identification of some of these as the causative variant. Further analyses in independent families and samples will be needed to assess if any of these mutations may be disease modifiers resulting in slight differences in the clinical presentation in these patients.

## Discussion

Through linkage analysis, homozygosity mapping and whole-exome sequencing, we identified a novel splice-site mutation in *ALS2* as a cause of a childhood-onset neurological disease with complex clinical presentation, thereby establishing a genetic diagnosis of juvenile-onset ALS in this family [13]. ALS comprises a heterogeneous group of rapidly progressing disorders leading to terminal neurodegeneration, involving both the upper and lower motor neurons. Only about 10% of ALS cases are familial while most cases of ALS present in sporadic forms (sALS). Familial ALS is genetically heterogeneous and many genes that harbor causal mutations have been identified [14], [15]. Most of the genes encode RNA processing proteins and aggregation of these abnormal proteins will lead to motor neuron damage resulting in disease [16]. However, the exact role of *ALS2* in disease etiology is still unclear [14].

Although juvenile ALS may be suspected based on the clinical symptoms alone, it requires a large number of clinical tests to draw the conclusion, since the presentation of *ALS2* mutation carriers is known to be heterogeneous and several other forms of familial spastic paraplegia or hereditary motor neuropathy may lead to similar presentations [11], [17], [18]. Recently, Sheerin and colleagues reported nonsense and frameshift mutations in *ALS2* in individuals presenting with generalized dystonia and cerebellar signs [17]. *ALS2* mutations are also known to cause complicated, infantile-onset forms of hereditary spastic paraplegia [11]. In this family, the first symptoms were the absence of speech and the inability to crawl normally, followed by severe scoliosis along with upper and lower motor symptoms. These patients have been living for more than 22–25 years since the first appearance of symptoms. MRI scans are usually normal in the ALS patients (www.ninds.nih.gov), but in these patients we did note thinning of the corticospinal tracts that may account for some of the upper motor neuron symptoms [19].

The identification of the *ALS2* mutation has firmly established the diagnosis of juvenile ALS in the family. This diagnosis was established using both linkage and sequencing which provided independent and unequivocal support for the causative role of this mutation in this family. Although whole-exome sequencing or sequencing of known neurological and neuromuscular disease genes in a single proband could also have led to the identification of this mutation [1], [20], it would also have picked up other rare mutations that are predicted to be damaging in these other genes. It is therefore important to understand the mode of inheritance of the disorder, and to confirm segregation in other family members to draw a firm conclusion based on these genetic results.

Identifying the underlying causative mutation can inform us of the specific and precise diagnosis, hence allowing us to provide better advice on potential therapies and prognosis in affected patients. It will also facilitate carrier or ante-natal testing and genetic counseling in unaffected members of the family, if desired. Finally, follow-up of these and other findings may lead to the identification of novel mechanisms of disease which may better develop our understanding of the pathophysiology of degenerative neurological disorders.

## Supporting Information

File S1 **Supporting information, containing Table S1, Table S2 and Text S1. Table S1**. 216 candidate genes for neurological diseases. **Table S2.** List of nonsynonymous variants identified in four exome-sequenced individuals that are rare (<1%) or absent in all 1000 genomes populations or the exome variant server database in 216 known neurological and neuromuscular disease genes. **Text S1.** Clinical Case Presentation.
(DOCX)
