# Supplementary material for: A Novel Splice-Site Mutation in ALS2 Establishes the Diagnosis of Juvenile Amyotrophic Lateral Sclerosis in a Family with Early Onset Anarthria and Generalized Dystonias
Source: PLoS One. 2014 Dec 4;9(12):e113258. doi: 10.1371/journal.pone.0113258 (PMC4256290; doi:10.1371/journal.pone.0113258)
Supplement: File S1 — Supporting information, containing Table S1, Table S2 and Text S1. Table S1. 216 candidate genes for neurological diseases. Table S2. List of nonsynonymous variants identified in four exome-sequenced individuals that are rare (<1%) or absent in all 1000 genomes populations or the exome variant server database in 216 known neurological and neuromuscular disease genes. Text S1. Clinical Case Presentation. (DOCX) [file pone.0113258.s001.docx]

**A novel splice-site mutation in *ALS2* establishes the diagnosis of juvenile amyotrophic lateral sclerosis in a family with early onset anarthria and generalized dystonias**

Saima Siddiqi et. al.

**File S1.** Supporting information, containing Table S1, Table S2 and Text S1.

**Table S1**. 216 candidate genes for neurological diseases

| **Disease** | **Candidate genes** | **Alternative names** |
| --- | --- | --- |
| **Hereditary Spastic Paraplegias** | *AFG3L2* |  |
| (Novarino et al 2014) | *ALS2* |  |
|  | *AMPD2* |  |
|  | *AP4B1* |  |
|  | *AP4E1* |  |
|  | *AP4M1* |  |
|  | *AP4S1* |  |
|  | *AP5Z1* |  |
|  | *ARG1* |  |
|  | *ARL6IP1* |  |
|  | *ARSI* |  |
|  | *ATL1* |  |
|  | *BICD2* |  |
|  | *BSCL2* |  |
|  | *C12ORF65* |  |
|  | *CCT5* |  |
|  | *CLN8* |  |
|  | *CYP2U1* |  |
|  | *CYP7B1* |  |
|  | *DDHD1* |  |
|  | *DDHD2* |  |
|  | *EIF2B5* |  |
|  | *ELOVL4* |  |
|  | *ENTPD1* |  |
|  | *ERLIN1* |  |
|  | *ERLIN2* |  |
|  | *FA2H* |  |
|  | *FLRT1* |  |
|  | *GAD1* |  |
|  | *GJA1* |  |
|  | *GJC2* |  |
|  | *HSPD1* |  |
|  | *KANK1* |  |
|  | *KIAA0196* |  |
|  | *KIF1A* |  |
|  | *KIF1C* |  |
|  | *KIF5A* |  |
|  | *L1CAM* |  |
|  | *MAG* |  |
|  | *MARS* |  |
|  | *MT-ATP6* |  |
|  | *NIPA1* |  |
|  | *NT5C2* |  |
|  | *PGAP1* |  |
|  | *PLP1* |  |
|  | *PNPLA6* |  |
|  | *RAB3GAP2* |  |
|  | *REEP1* |  |
|  | *REEP2* |  |
|  | *RTN2* |  |
|  | *SLC16A2* |  |
|  | *SLC33A1* |  |
|  | *SPAST* |  |
|  | *SPG11* |  |
|  | *SPG20* |  |
|  | *SPG21* |  |
|  | *SPG7* |  |
|  | *TECPR2* |  |
|  | *USP8* |  |
|  | *VCP* |  |
|  | *VPS37A* |  |
|  | *WDR48* |  |
|  | *ZFR* |  |
|  | *ZFYVE26* |  |
| **Charcot-Marie-Tooth disease** | *AARS* | *CMT2N* |
|  | *CMT2G* |  |
|  | *CMTDIA* |  |
|  | *CMTX2* |  |
|  | *CMTX3* |  |
|  | *DHTKD1* | *KIAA1630,AMOXAD,CMT2Q,* |
|  | *DNM2* | *CMTDIB,CMTDI1,CMT2M,LCCS5,* |
|  | *DYNC1H1* | *DNCL,DNECL,CMT20,MRD13,SMALED1,* |
|  | *EGR2* | *KROX20* |
|  | *FGD4* | *FRABIN,CMT4H,* |
|  | *FIG4* | *KIAA0274,SAC3,ALS11,YVS,* |
|  | *GARS* | *SMAD1,CMT2D,HMN5,* |
|  | *GDAP1* | *CMT4A,CMT2K,CMTRIA,* |
|  | *GJB1* | *CX32,CMTX1,* |
|  | *GNB4* | *CMTD1F* |
|  | *HOXD10* | *HOX4D* |
|  | *HSPB1* | *HSP27,CMT2F,HMN2B,* |
|  | *HSPB8* | *H11,E2IG1,DHMN2,CMT2L,HMN2A,* |
|  | *INF2* | *FSGS5,C14orf173,CMTDIE,* |
|  | *KARS* | *CMTRIB,DFNB89,* |
|  | *KIF1B* | *CMT2A,CMT2A1,NBLST1,* |
|  | *LITAF* | *CMT1C* |
|  | *LMNA* | *LMN1,EMD2,FPLD2,CMD1A,HGPS,LGMD1B* |
|  | *MED25* | *PTOV2,ARC92,CMT2B2,* |
|  | *MFN2* | *KIAA0214,CMT2A2,* |
|  | *MPZ* | *CMT1B,CMTDID,CHM,DSS,* |
|  | *MTMR2* | *CMT4B1* |
|  | *NDRG1* | *HMSNL,CMT4D,* |
|  | *NEFL* | *CMT2E,CMT1F,* |
|  | *PDK3* | *CMTX6* |
|  | *PLEKHG5* | *KIAA0720,DSMA4,CMTRIC,* |
|  | *PMP22* | *CMT1A,CMT1E,DSS,* |
|  | *PRPS1* | *CMTX5,DFNX1,DFN2,* |
|  | *PRX* | *CMT4F* |
|  | *RAB7* | *CMT2B,PSN,* |
|  | *SBF1* | *MTMR5,CMT4B3,* |
|  | *SBF2* | *MTMR13,CMT4B2,* |
|  | *SH3TC2* | *KIAA1985,MNMN,* |
|  | *TRIM2* | *KIAA0517,CMT2R,* |
|  | *YARS* | *CMTDIC,TYRRS,YTS,YRS,* |
| **Amyotrophic lateral sclerosis** | *DCTN1* | *HMN7B* |
|  | *PRPH* |  |
|  | *SOD1* | *ALS1* |
|  | *NEFH* |  |
|  | *TRPM7* | *LTRPC7,CHAK,ALSPDC,* |
|  | *C9orf72* | *FTDALS,ALSFTD,* |
|  | *VCP* | *IBMPFD1,ALS14,* |
|  | *ATXN2* | *ATX2,SCA2,ASL13,* |
|  | *ALS2* | *ALSJ,PLSJ,IAHSP,* |
|  | *UBQLN2* | *PLIC2,CHAP1,ALS15,* |
|  | *MAPT* | *MTBT1,DDPAC,MSTD,* |
|  | *SETX* | *SCAR1,AOA2,ALS4,* |
|  | *FUS* | *TLS,ALS6,ETM4,* |
|  | *VAPB* | *VAPC,ALS8,* |
|  | *ANG* | *RNASE5,ALS9,* |
|  | *TARDBP* | *TDP43,ALS10,* |
|  | *FIG4* | *KIAA0274,SAC3,ALS11,YVS,* |
|  | *OPTN* | *GLC1E,FIP2,HYPL,NRP,ALS12,* |
|  | *VCP* | *IBMPFD1,ALS14,* |
|  | *SIGMAR1* | *SRBP,ALS16,* |
|  | *CHMP2B* | *DMT1,VPS2B,ALS17,* |
|  | *PFN1* | *ALS18* |
|  | *HNRNPA1* | *IBMPFD3,ALS19,* |
| **Spinal muscular atrophy** | *ASAH1* | *AC,SMAPME,* |
|  | *ATP7A* | *MNK,MK,OHS,SMAX3,* |
|  | *BICD2* | *KIAA0699,SMALED2,* |
|  | *DNAJB2* | *HSJ1,HSPF3,DSMA5,* |
|  | *DYNC1H1* | *DNCL,DNECL,CMT20,MRD13,SMALED1,* |
|  | *PLEKHG5* | *KIAA0720,DSMA4,CMTRIC,* |
|  | *SMAJ* |  |
|  | *SMAR* |  |
|  | *SMN1* | *SMA1,SMA2,SMA3,SMA4,* |
|  | *SMN2* |  |
|  | *TRPV4* | *VROAC,HMSN2C,CMT2C,SPSMA,SSQTL1,SMAL* |
|  | *UBA1* | *UBE1,GXP1,A1ST,SMAX2,AMCX1,* |
|  | *VAPB* | *VAPC,ALS8,* |
| **Ataxias** | *ABCB7* | *ABC7,ASAT,* |
|  | *ABHD12* | *PHARC* |
|  | *ANO10* | *TMEM16K,SCAR10,* |
|  | *APTX* | *AOA,AOA1,* |
|  | *ATCAY* | *CLAC,KIAA1872,* |
|  | *ATM* | *ATA,AT1,* |
|  | *ATP2B3* | *PMCA3,SCAX1,* |
|  | *ATP8A2* | *ATPIB,CAMRQ4,* |
|  | *ATR* | *FRP1,SCKL1,FCTCS,* |
|  | *ATXN1* | *ATX1,SCA1,* |
|  | *ATXN10* | *SCA10* |
|  | *ATXN7* | *SCA7,OPCA3,* |
|  | *ATXN8* |  |
|  | *ATXN8OS* | *SCA8,KLHL1AS,* |
|  | *BEAN* | *SCA31* |
|  | *CA8* | *CALS,CARP,CAMRQ3,* |
|  | *CACNB4* | *EJM6,EA5,EIG9,* |
|  | *CAMTA1* | *KIAA0833,CANPMR,* |
|  | *CP* |  |
|  | *DNMT1* | *MCMT,HSN1E,ADCADN,* |
|  | *EA3* |  |
|  | *EA7* |  |
|  | *FGF14* | *FHF4,SCA27,* |
|  | *FLVCR1* | *AXPC1,PCARP,* |
|  | *FRDA2* |  |
|  | *FXN* | *FRDA,FARR,X25,* |
|  | *GRM1* | *MGLUR1,GRM1A,SCAR13,* |
|  | *ITPR1* | *SCA15,SCA16,SCA29,* |
|  | *KCNA1* | *AEMK,EA1,* |
|  | *KCNC3* | *SCA13* |
|  | *MRE11A* | *MRE11,ATLD,* |
|  | *MTPAP* | *PAPD1,SPAX4,* |
|  | *NPAT* | *E14* |
|  | *PDYN* | *SCA23* |
|  | *PIK3R5* | *p101* |
|  | *RNF170* | *SNAX1* |
|  | *RNF216* | *TRIAD3,ZIN,CAHH,* |
|  | *SACS* | *ARSACS* |
|  | *SCA18* | *SMNA* |
|  | *SCA19* |  |
|  | *SCA20* | *DUP11q12,C11DUPq12,* |
|  | *SCA21* |  |
|  | *SCA25* |  |
|  | *SCA26* |  |
|  | *SCA30* |  |
|  | *SCA32* |  |
|  | *SCA34* |  |
|  | *SCA4* |  |
|  | *SCAR12* |  |
|  | *SCAR2* | *CPD3* |
|  | *SCAR3* | *SCABD* |
|  | *SCAR4* | *SCASI* |
|  | *SCAR6* | *CLA3* |
|  | *SCAR7* |  |
|  | *SCAX1* | *CLA2,OPCA,* |
|  | *SCAX5* |  |
|  | *SCN8A* | *CIAT,EIEE13,* |
|  | *SETX* | *SCAR1,AOA2,ALS4,* |
|  | *SLC1A3* | *EAAT1,EA6,* |
|  | *SPAX1* |  |
|  | *SPAX2* |  |
|  | *SPAX3* | *ARSAL* |
|  | *SPTBN2* | *SCA5,SCAR14,* |
|  | *SYNE1* | *KIAA0796,KIAA1756,KIAA1262,SCAR8,EDMD4,* |
|  | *SYT14* | *SCAR11* |
|  | *TDP1* |  |
|  | *TGM6* | *TG6,TGY,SCA35,* |
|  | *TTBK2* | *SCA11* |
|  | *TTPA* | *TTP1,AVED,* |
|  | *WDR81* | *CAMRQ2* |
|  | *ZNF592* | *KIAA0211,SCAR5,CAMOS,* |
| **Dystonia** | *ACTB* | *BRWS1* |
|  | *ANO3* | *TMEM16C,C11orf25,DYT24,* |
|  | *DRD5* | *DRD1B,DRD1L2,* |
|  | *DYT13* |  |
|  | *DYT15* |  |
|  | *DYT21* |  |
|  | *DYT23* |  |
|  | *DYT7* |  |
|  | *EPRPDC* |  |
|  | *GNAL* | *DYT25* |
|  | *SCP2* |  |
|  | *SLC2A1* | *GLUT1,DYT18,PED,GLUT1DS,EIG12,DYT9* |
|  | *THAP1* | *DYT6* |
|  | *TUBB4A* | *TUBB4,TUBB5,DYT4,HLD6,* |

**Table S2.** List of nonsynonymous variants identified in four exome-sequenced individuals that are rare (<1%) or absent in all 1000 genomes populations or the exome variant server database in 216 known neurological and neuromuscular disease genes (Table S1). Hom=homozygous, Het=heterozygous.

| **Gene Name** | **Amino Acid Substitution** | **SIFT Prediction** | **dbSNP Id** | **IV-6 (case)** | **IV-9 (case)** | **III-2 (control)** | **III-5 (control)** |
| --- | --- | --- | --- | --- | --- | --- | --- |
| *CAMTA1* | V300I | TOLERATED | novel | - | - | - | Het |
| *TARDBP* | L5P | TOLERATED | rs61730366 | - | Het | - | Het |
| *MTPAP* | I23L | DAMAGING, Low confidence. | novel | - | Het | - | - |
| *AARS* | N944K | TOLERATED | novel | Het | - | Het | - |
| *KIF1C* | Q434H | TOLERATED | rs144939400 | Het | - | Het | Het |
| ***ALS2*** | **splice** | **NA** | **novel** | **Hom** | **Hom** | **Het** | **Het** |
| *SH3TC2* | V222I | TOLERATED | novel | Hom | - | Het | Het |
| *GARS* | G546E | DAMAGING | novel | - | Het | - | Het |
| *KANK1* | G12D | TOLERATED | novel | - | - | - | Het |
| *TTPA* | splice | NA | novel | Hom | Hom | Hom | Hom |

**Text S1.** Clinical Case Presentation

The affected family is of Pakistani descent. Two siblings in family A and two in family B were affected.


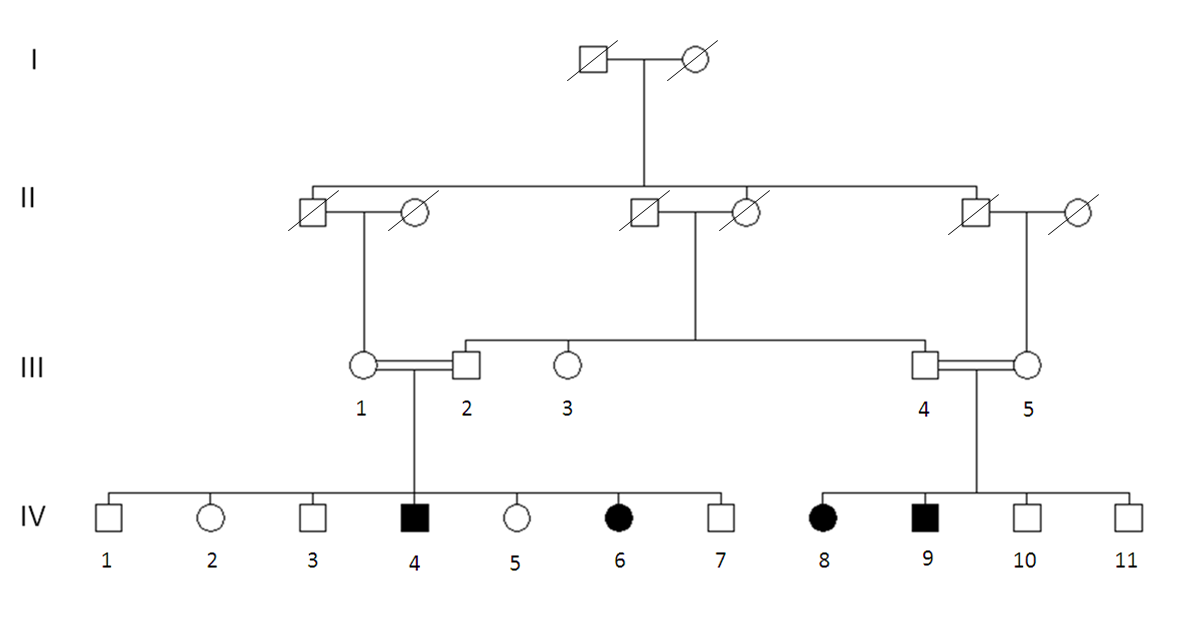


**Family A**

**IV-4** is a 21 year old male. He had delayed motor milestones with neck holding at 10 months of age and crawling at 1 year. He was never able to walk and required a wheelchair from age of 5 years. Later on, worsening of muscle weakness and development of thoracic scoliosis at age of 9 confined him to bed. He presented to our hospital at the age of 21. At that time he was anarthric, had marked muscle atrophy, decreased muscle tone and power with dystonic posturing of hands and neck. Reflexes were brisk with clonus and flexor planter response. His cognition and bladder control was intact. Response to commands was by movement of his eyes.

**IV-6** is a 24 year old female. She had abnormal early milestones including poor neck holding and delayed crawling. She never managed to walk independently. Vocalization of single words was reported at 4 years of age but later on she stopped talking by the age of 7 years. Because of early and severe development of thoracolumbar scoliosis and progressive muscle weakness, she was bed-bound since the age of 5 years. At the time of examination, she had generalized muscles wasting more in legs, right foot drop, severe scoliosis and facial grimace. Global muscular weakness was evident upon examination with brisk reflexes and bilateral flexor planter response. Cognitive decline was only mild and her bowel and bladder control was adequate.

**Family B**

**IV-8** is a 22 year old female with history of delayed motor mile stones. She had weakness initially in her lower limbs and started walking with support at age of 4 years. Weakness progressed gradually to upper limbs along with development of thoracic scoliosis leading her to be wheel chair bound by age of 6 years. She was brought to our hospital at 22 years of age and her examination revealed anarthria, drooling from mouth, generalized wasting of muscles and hypotonia, and uncontrollable laughter. Her orientation and cognition was generally intact and she was able to follow one-step commands and responded with eye movements. Muscle power in all 4 limbs was of grade 1. Bilateral extensor planer response was observed along with brisk reflexes.

**IV-9** was able to achieve normal motor milestones up to 1 year of age when he started walking with support. Rapidly progressive weakness of muscles and development of very early scoliosis affecting mainly the thoracic spine limited his mobility and he was contained to a wheelchair by 3^rd^ year of his life. By 19 years of age, he was bed-bound. At the time of assessment, he was 21 years old with anarthria, emotional liability, severe scoliosis, opisthotonus, contractures in upper and lower limbs and generalized dystonias. He had pathologically brisk reflexes and bilateral extensor planter response. Cranial nerve examination was unremarkable. Language reception was preserved and bower/bladder control was intact.
